# Practices and Attitudes of Surgeons With Regard to Spilled Gallstones During Laparoscopic Cholecystectomy: A Cross-Sectional Study From Saudi Arabia

**DOI:** 10.7759/cureus.53115

**Published:** 2024-01-28

**Authors:** Mohammed Alfehaid, Moath Aljohani, Sajad A Salati, Shoug Alaodah, Wejdan Alresheedi, Raghad Almarshud

**Affiliations:** 1 Department of Surgery, Unaizah College of Medicine and Medical Sciences, Qassim University, Unaizah, SAU; 2 Department of Family and Community Medicine, Unaizah College of Medicine and Medical Sciences, Qassim University, Unaizah, SAU

**Keywords:** malignancy, granuloma, abscess, complications, laparoscopic cholecystectomy, spilled gallstones

## Abstract

Background: Gallbladder perforation and gallstone leakage are frequent complications following laparoscopic cholecystectomy (LC). Failure to remove gallstones may result in several issues that manifest immediately or years later. The goal of this study was to evaluate the attitudes of surgeons and the procedures used by them to deal with gallstone spillage during LC.

Methods: A cross-sectional design was followed. Surgeons in nine healthcare facilities in the Qassim region of Saudi Arabia were approached through non-probability convivence sampling and the survey was distributed in each of the general surgery divisions. The study included general surgeons who currently performed LC and incomplete responses and interns were excluded. A self-administered questionnaire was developed with 18 questions regarding demographics, center, and designation at the hospital, surgeons’ experience of LC, and exposure to gallstone spillage. Furthermore, items regarding knowledge, attitude, and self-reported practices related to gallstone spillage such as incidence, complications, and intervention taken to prevent gallstone spillage were also included. The level of significance was set at P <0.05.

Results: There were 82 participants of both genders, including consultants, specialists, and residents. While only 23 (28%) participants had actually observed patients with complications from spilled stones, 46 (56.1%) participants were aware of this possibility, 53 (64.6%) deemed it inappropriate to bring up gallstone spillage when securing consent for LC, and 67 (81.7%) believed that such an incident needed to be documented in the operation notes. Only 11 (13.4%) thought that the complications arising out of the unretrieved gallstones should fall under the legal purview of the operative surgeon. There were very few complications of spilled gallstones that the participants were aware of, and none of them anticipated problems to arise more than three years after LC.

Conclusions: Awareness of the risks associated with gallstone spillage during LC needs to be raised, and it is imperative to standardize the practices related to their management.

## Introduction

Laparoscopic cholecystectomy (LC) has become the gold standard for surgical treatment of symptomatic gallstone disease because of many proven advantages, including reduced postoperative discomfort, shorter hospital stays, faster recovery, and improved cosmetic outcomes [1]. However, it's associated with an array of specific complications, one of which is gallstone spillage. This may present intensely in the early postoperative phase as surgical site pain and infections, or it may take a long time to appear as a range of apparently unrelated symptoms that are difficult to diagnose [2,3]. The attitudes, expertise, and practices of surgeons with relation to the prevention and management of gallstone spillages have not been extensively studied, and the few studies available in the literature point towards gaps in the knowledge of surgeons related to potential complications [4]. Furthermore, the practices regarding the reporting and management of gallstone spillage are widely different due to the apparent lack of uniform evidence-based protocols [4]. So far, no research has been undertaken in Saudi Arabia to look into surgeons' perceptions and practices related to gallstone spillage. In this context, an observational study was conducted in the Al-Qassim region of Saudi Arabia to explore the prevailing practices and attitudes of the practicing surgeons with regard to spilled gallstones.

## Materials and methods

Study design and setting

This was a cross-sectional, observational study. The study was facility-based and conducted from October 2022 to December 2022 at the surgical departments of nine healthcare facilities in the Qassim region of Saudi Arabia: King Saud Hospital Unaizah, King Fahad Specialty Hospital Buraidah, Buraidah Central Hospital, Qassim University Medical City, Armed Forces Hospital, Al Qassim National Hospital, Hayat National Hospital, Al-Rass General Hospital, and Dr. Sulaiman Al Habib Hospital. The study was duly approved by the Regional Ethics Committee in the Health Directorate of Al Qassim region of Saudi Arabia vide letter number N-607/44/3031, on September 28, 2022.

Sample size and sampling technique

The sample size was calculated based on the assumption of having 10 participants per predictor as per Peduzzi et al. [5]. We had six possible predictors thereby accounting for a sample size of 60 and we added 10% to account for incompleteness and non-responses, thus arriving at a sample of 66 participants [5]. We followed the non-probability convenience sampling technique and all surgeons who were on duty during the data collection were invited to participate in our study. The inclusion criteria were general surgeons who are currently doing laparoscopic cholecystectomies and the exclusion criteria were incomplete responses and interns.

Data collection methods

An anonymous questionnaire was provided to the participants. It comprised 18 items including demographics such as age, gender, and nationality. The second section included items related to the center the surgeons worked in and their designation (consultant, specialist, or resident). The third section covered items related to LC experience and their previous exposure to spilled stones incidents during cholecystectomy. The last section included questions on knowledge, attitude, and practices regarding gallstone spillage: incidence of gallstone spillage during laparoscopic surgery, whether leaving spilled gallstones outside the biliary system may harm the patient, if they agreed with the hypothesis that says that the body defence has a role in protecting the patient from developing complications following spilled gallstones, whether information about spilled stones should be included in the consent form, and if they ever witnessed a complication of spilled stones. Additionally, they were asked to choose from a list which complications of spilled stones were more prevalent and what kind of intervention they would take if they encountered a spilled stone. Furthermore, questions were included that explored their opinion regarding documentation of gallstone spillage in operative notes, the optimum duration of follow-up after gallstone spillage, and the legal liability of surgeons if complications of gallstone spillage ever happened. Lastly, a final open-ended question asked whether they preferred certain techniques during the surgery to prevent gallstone spillage. The questionnaire items were developed based on previous studies and reviewed by consultants in general surgery and preventive medicine to ensure face and content validity [4,6].

The study's goals and objectives were clarified to the participants along with the time needed for completion, and if they agreed to take part, they were asked to sign a consent form. A pilot study was undertaken after enrolling nine participants to assess the suitability and clarity of the questionnaire and estimate the time required for data collection.

Statistical analysis

Analyses were carried out using IBM SPSS Statistics for Windows, Version 22.0 (Released 2013; Armonk, New York, United States). Descriptive analyses were carried out and the results were presented using frequencies and proportions (for categorical data) and mean and standard deviation for numerical continuous data. Inferential analyses were conducted using the unpaired t-test to assess differences in average age between those who indicated no intervention and any intervention to manage gallstone spillage. Also, the chi-square test was used to determine the presence and strength of any associations between the categorical demographic variables and participant’s choice to adopt the options of ‘no intervention’ or ‘intervention’ to manage gallstone spillage. The level of significance was set as <0.05.

## Results

Demographic distribution of the participants

Eighty-three individuals submitted the questionnaires. Of these, one was incomplete, and hence, a total of 82 participants were included in the study with a mean age of 38.7 (SD 1.2) (Table 1). The participants included 36 consultants (43.9%) followed by 23 (28.0%) specialists and 23 (28.0%) residents pursuing postgraduate training in General Surgery. There were 73 males (89.0%) and nine females (11.0%). Forty-nine (59.8%) participants were of Saudi nationality and the rest (n=33; 40.2%) were non-Saudis. Most of the participants belonged to the three major referral hospitals of the Qassim region, namely King Saud Hospital Unaizah (n=26; 31.7%), King Fahad Specialty Hospital (n=23; 28.0%), and Buraidah Central Hospital (n=18; 22.0%).

**Table 1:** Demographic distribution of the participants (N = 82)

Variables	Frequency	Percentage (%)
Age (years), Mean ± SD	38.7 ± 1.2	
Designation		
Consultant	36	43.9
Specialist	23	28.0
Resident	23	28.0
Gender		
Male	73	89.0
Female	9	11.0
Nationality		
Saudi	49	59.8
Non-Saudi	33	40.2
Hospital center		
King Saud Hospital Unaizah	26	31.7
King Fahad Specialty Hospital	23	28.0
Buraidah Central Hospital	18	22.0
Qassim University Medical City	5	6.1
Armed Forces Hospital	3	3.7
Al Qassim National Hospital	2	2.4
Hayat National Hospital	2	2.4
Al Rass General Hospital	2	2.4
Dr. Sulaiman Al Habib Hospital	1	1.2

Experiences of gallstone spillage

Forty-two (51.2%) participants had conducted up to 50 LCs in the preceding year (Table 2), and 14 (17.1%) had participated in more than 100 LCs during the same period. Of the participants, 77 (93.9%) had encountered gallstone spillage during LC, while 59 (72%) believed the incidence of gallstone spillage during LC to be 10% or less and another 22.0% thought the incidence was 11-20%.

**Table 2:** Participants’ personal experiences of gallstone spillage (N = 82)

Variables	Frequency	Percentage (%)
Number of laparoscopic cholecystectomies performed		
0-25	19	23.2
26-50	23	28.0
51-75	10	12.2
76-100	16	19.5
>100	14	17.1
Ever encountered gallstone spillage		
Yes	77	93.9
No	5	6.1
Encountered patient with complications from spilled gallstone		
Yes	23	28.0
No	59	72.0
Complications of spilled stones encountered by participants who replied in affirmative to the above question (n=23)		
Port-site infection	7	30.4
Intra-abdominal infection/abscess	7	30.4
Chronic pain	5	21.7
Inflammatory omental mass	1	4.3
Bowel adhesion	1	4.3
Port-site sinus formation	1	4.3
Biliary tree obstruction due to extraluminal bile duct obstruction	1	4.3

Participants’ perceptions regarding gallstone spillage

Gallstone spillage in LC could happen in up to 10% of cases, according to 59 participants (72%), and in up to 20% of cases according to 18 individuals (22%) (Table 3). Thirty-six (43.9%) participants considered the unretrieved spilled gallstones harmless, whereas 46 (56.1%) believed they may adversely affect the patient. With respect to the requirement for informed consent, the majority (64.6%) argued that it was not necessary to include the possibility of gallstone spilling. Regarding complications resulting from unretrieved gallstones, only 23 (28%) of the participants had actually seen such patients, and 59 (72%) had not seen any in their career. Among these 23 participants who had encountered patients with complications, the major complications included port-site infection (n = 7; 30.4%), intra-abdominal infection or abscess (n = 7; 30.4%), and chronic pain (n = 5; 21.7%).

**Table 3:** Participants’ perceptions regarding gallstone spillage (N = 82)

Variables	Frequency	Percentage (%)
Perceived incidence of gallstone spillage		
≤ 10%	59	72.0
≤ 20%	18	22.0
≤30%	2	2.4
≤40%	3	3.7
Leaving spilled gallstone may harm the patients in some way		
Yes	46	56.1
No	36	43.9
Potential complications of spilled gallstones that you are aware of:		
Port-site infection	63	76.8
Omental granuloma	21	25.6
Peritonitis/ Intraabdominal abscess	16	19.5
Intestinal obstruction	7	8.5
Sepsis	4	4.9
Pain	1	1.2
Inclusion of gallstone spillage in informed consent		
Yes	29	35.4
No	53	64.6

Interventions and techniques for managing gallstone spillage

Seventy-nine (96.3%) participants indicated using instruments (n=61; 74.4%) and/or peritoneal wash and suction (n=46; 56.1%) as a means of intervening for gallstone spilling. Three (3.7%) individuals, however, stated that they would not conduct any kind of intervention. sixty-seven (81.7%) participants agreed that the documentation of gallstone spillage in operation notes is important, but 15 (18.3%) participants either felt that documentation was not required or were unsure.

Of the participants, 60 (73.2%) felt that a follow-up visit was adequate for patients who had spilled gallstones, and 15 (18.3%) stated they would follow up with patients for six months following the incident. Only six (7.3%) would follow up for one year. Out of all the participants, only 11 (13.4%) thought that the operating surgeon might be held legally responsible for any consequences that resulted from spilling gallstones. The remaining (n=24; 29.3%) were either unsure or disagreed (n=47; 57.3%).

With regard to favored methods for averting gallstone spillage, 35 (42.7%) participants opted for cautious dissection combined with adequate exposure. Utilizing endobags to remove the gallbladder was another technique mentioned by 19 (23.2%) responders (Table 4).

**Table 4:** Interventions and techniques for managing gallstone spillage (N = 82)

Variables	Frequency	Percentage (%)
Intervention to manage gallstone spillage		
No intervention	3	3.7
Convert to open retrieval	0	0.0
Laparoscopic retrieval with instruments	61	74.4
Peritoneal wash and suction	46	56.1
Other	0	0.0
Necessary to document gallstone spillage in operation notes		
Yes	67	81.7
No	9	11.0
I don't know	6	7.3
Duration needed for follow-up for gallstone spillage patients		
One visit	60	73.2
6 months	15	18.3
1 year	6	7.3
3 years	1	1.2
Operating surgeons can be held legally liable for complications following gallstone spillage		
Yes	11	13.4
No	47	57.3
I don't know	24	29.3
Preferred techniques to prevent gallstone spillage		
Usage of endobag for gallbladder retrieval	19	23.2%
Good exposure with careful dissection	26	42.7%
Usage of endogauze at the hepato-renal recess to collect gallstone	3	4.9%
Decrease the current of diathermy during dissection	2	4.9%
Aspiration of the distended gallbladder	3	3.7%
Increase the port size	1	2.4%
No special technique	24	29.3%

Concerning the variables impacting the participants' selection of intervention or otherwise to handle gallstone spillage, it was shown that there was no statistically significant correlation between any demographic characteristic and the choices made by the participants (Table 5). Furthermore, no statistically significant correlation was observed between the decision of the participants to not perform any intervention and their prior experience in managing patients with complications of gallstone spillage (p > 0.05). Younger participants, whose mean age was 31 years, stated they would use no intervention at all, but older participants, whose mean age was 39 years, claimed they would use some sort of intervention (p = 0.201).

**Table 5:** Univariate analyses exploring associations between demographic characteristics and interventions to manage gallstone spillage (N = 82) SD: Standard deviation

Variables	N (%)			p-value
	No intervention	Any intervention	Total	
Age (years), Mean ± SD	31.0 ± 7.0	39.0 ± 10.6	NA	0.201
Designation				
Consultant	1 (2.8%)	35 (97.2%)	36 (100.0%)	0.458
Specialist	0 (0.0%)	23 (100.0%)	23 (100.0%)	
Resident	2 (8.7%)	21 (91.3%)	23 (100.0%)	
Gender				
Male	2 (2.7%)	71 (97.3%)	73 (100.0%)	0.207
Female	1 (11.1%)	8 (88.9%)	9 (100.0%)	
Nationality				
Saudi	3 (6.1%)	46 (93.9%)	49 (100.0%)	0.148
Non-Saudi	0 (0.0%)	33 (100.0%)	33 (100.0%)	
Hospital center				
King Fahad Specialty Hospital	2 (8.7%)	21 (91.3%)	23 (100.0%)	0.940
Buraidah Central Hospital	0 (0.0%)	18 (100.0%)	18 (100.0%)	
Dr. Sulaiman Al Habib Hospital	0 (0.0%)	1 (100.0%)	1 (100.0%)	
Al Qassim National Hospital	0 (0.0%)	2 (100.0%)	2 (100.0%)	
Qassim University Medical City	0 (0.0%)	5 (100.0%)	5 (100.0%)	
Armed Forces Hospital	0 (0.0%)	3 (100.0%)	3 (100.0%)	
King Saud Hospital Unaizah	1 (3.8%)	25 (96.2%)	26 (100.0%)	
Hayat National Hospital	0 (0.0%)	2 (100.0%)	2 (100.0%)	
Al Rass General Hospital	0 (0.0%)	2 (100.0%)	2 (100.0%)	
Encountered patient with complication from gallstone spillage				
Yes	0 (0.0%)	23 (100.0%)	23 (100.0%)	0.556
No	3 (5.1%)	56 (94.9%)	59 (100.0%)	

Similarly, when testing for associations between demographic variables and experience of handling patients with complications from spilled gallstones, there was no statistically significant association recorded (p > 0.05) (Table 6).

**Table 6:** Univariate analyses exploring associations between demographic characteristics and experience of gallstone spillage (N = 82) SD: Standard deviation

Variables	Handled patient with complications from spilled gallstone, N (%)			p-value
	Yes	No	Total	
Age (years), Mean ± SD	36.6 ± 9.3	39.5 ± 11.0	NA	0.292
Designation				
Consultant	9 (25.0%)	27 (75.0%)	36 (100.0%)	0.863
Specialist	7 (30.4%)	16 (69.6%)	23 (100.0%)	
Resident	7 (30.4%)	16 (69.6%)	23 (100.0%)	
Gender				
Male	22 (30.1%)	51 (69.9%)	73 (100.0%)	0.433
Female	1 (11.1%)	8 (88.9%)	9 (100.0%)	
Nationality				
Saudi	14 (28.6%)	35 (71.4%)	49 (100.0%)	0.898
Non-Saudi	9 (27.3%)	24 (72.7%)	33 (100.0%)	
Hospital center				
King Fahad Specialty Hospital	10 (43.5%)	13 (56.5%)	23 (100.0%)	0.370
Buraidah Central Hospital	3 (16.7%)	15 (83.3%)	18 (100.0%)	
Dr. Sulaiman Al Habib Hospital	0 (0.0%)	1 (100.0%)	1 (100.0%)	
Al Qassim National Hospital	1 (50.0%)	1 (50.0%)	2 (100.0%)	
Qassim University Medical City	2 (40.0%)	3 (60.0%)	5 (100.0%)	
Armed Forces Hospital	1 (33.3%)	2 (66.7%)	3 (100.0%)	
King Saud Hospital Unaizah	5 (19.2%)	21 (80.8%)	26 (100.0%)	
Hayat National Hospital	0 (0.0%)	2 (100.0%)	2 (100.0%)	
Al Rass General Hospital	1 (50.0%)	1 (50.0%)	2 (100.0%)	

## Discussion

In performing cholecystectomies for a variety of indications, such as acute or chronic cholecystitis, symptomatic cholelithiasis, biliary dyskinesia, acalculous cholecystitis, gallstone pancreatitis, and gallbladder masses or polyps, LC has emerged as the gold standard, due to lower incidence of postoperative morbidity and early recovery [7,8]. Nevertheless, there are acknowledged complications associated with LC, just as there are with other surgical techniques [9]. Gallstone spillages and iatrogenic gallbladder perforations happen frequently; the former can happen as often as 10-40% of the time [10,11] and the latter as often as 6-40% of the time [12-14].

In our study, 77 (93.9%) participants had encountered gallstone spillage during LC in their careers, and 59 (72%) believed the incidence of gallstone spillage during LC to be 10% or less. However, one worrisome figure we found is that 36 (43.9%) participants believed that unretrieved gallstones do not potentially lead to any complications. In contrast, in a similar study by Khan et al. in 2013, 83% of respondents were found to be aware of the possibility of complications from lost gallstones as opposed to only 10% who believed that unretrieved gallstones cannot cause complications [6].

Another significant area of deficient awareness that was detected in our study is related to the timeframe for the emergence of complications due to spilled gallstones. Sixty (73.2%) participants indicated that a single visit was sufficient for follow-up of patients and 15 (18.3%) indicated that they would continue to follow up with patients for six months after the incident of the spillage; only six (7.3 %) and one (1.2%) reported that they would follow up till one year and three years, respectively. In contrast, Khan et al. found that only 28% of participants in their study weren't fully aware of the timeframe for presentation of complications and 15% of the participants believed complications to arise even beyond five years of LC [6]. In a similar study conducted by Yethadka et al. in 2014, 76.8% of the participants thought that two years were sufficient to follow up after spilled stones and that they had no knowledge about the possibility of delayed complications [4]. In the literature, complications from unretrieved gallstones have been described as occurring years or even decades later [15-17]. Morris et al. reported a 71 year-old-female who presented with complete bowel obstruction 15 years after LC [18]. Exploratory laparotomy revealed cholesterol gallstones throughout the abdomen and many calculi were found embedded within a dense mesenteric cicatrix causing ileocolic volvulus. Ladic et al. reported a 54-year-old male who presented with persistent hemoptysis 10 years after LC [19]. Imaging revealed a mass in the upper part of the left kidney spreading directly into the adjacent diaphragm and left lower lobe of the lung. Laparotomy was undertaken and a wide excision of the mass was undertaken along with excision of the infiltrated part of the left hemidiaphragm, splenectomy, and decortication of the left lung. On analysis of the mass, of a gallstone was found that over years had initiated an inflammatory process with dense fibrosis. Mehmood et al. reported a 73-year-old female who suffered from acute abdominal pain 10 years after LC and evaluation had revealed two radiopaque gallstones causing a right lateral abdominal wall abscess [20]. Frade et al. reported a 78-year-old male who suffered from recurrent episodes of abdominal pain with fluctuating fever [21]. The complaint started one year after LC and it was only during one of the severe episodes after 10 years, that a detailed evaluation led to the discovery of unretrieved gallstones causing right retroperitoneal and subhepatic collection.

Proper reporting of gallstone spilling in operating room notes was deemed essential by 67 (81.7%) participants, but 15 (18.3%) either disagreed or were unsure. However, the majority of the participants (n = 53; 64.6%) felt that it is not important to mention the possibility of gallstone spillage while securing the consent for LC. This is in line with other similar studies in the literature that have identified the absence of documentation of dropped stones as an area of concern. In the study by Khan et al., 88% of participants believed that documentation of lost gallstones should be done although only 70% of participants reported that they did so in actual practice and only 41% of participants actually informed patients of spilled gallstones postoperatively [6]. In the survey conducted by Mullerat et al., it was found that only 50% of the operating surgeons actually informed patients about lost gallstones [22]. In the study by Yethadka et al., around 80% of participants agreed that stone spillage should be mentioned in the operative notes but again only 70% practiced the same [4].

On the question of legal liabilities of the operating surgeon for complications following gallstone spillage, only 11 (13.4%) participants believed that the operating surgeon can be held legally liable and the majority either disagreed (n=47; 57.3%) or else were not sure (n=24; 29.3%). In the study by Yethadka et al., 76% of the participants disagreed and only 24% felt that the surgeons ought to carry legal liabilities [4].

Khan et al., in their study, presented 18 potential complications of lost stones and only 20% of the participants identified more than eight complications for which they can consider lost gallstones causal [6]. In our study, we presented fewer complications and left it as an open-ended question to register the complications. Accordingly, only a few complications were mentioned including port-site infection, omental granuloma, peritonitis/ intraabdominal abscess, intestinal obstruction, sepsis, and pain, indicating thereby that participants are not fully aware of the variable nature of these complications. An important complication that was not mentioned by any participant was the possibility of diagnostic difficulties with lesions mimicking serious disorders like cancer. Capolupo et al. reported a 73-year-old who, after one year of LC, in whom imaging for urinary symptoms revealed multiple peritoneal nodules mimicking neoplastic nodules [23]. Jeong et al. reported an asymptomatic 59-year-old male in whom an ill-defined mass lesion in the right subhepatic space was detected incidentally in a CT scan, three months after LC [24]. Positron emission tomography (PET) scan displayed hypermetabolism but no stone was visualized on imaging, nor was there any mention of stone spillage in opera­tive notes. A provisional diagnosis of peritoneal metastasis was made, and wedge resection of the liver, wedge resection of the transverse colon, and omentectomy were undertaken. However, histopathology of the resected specimen revealed a foreign body granuloma due to gallstone. Suarez-Zamora et al. reported a 29-year-old female in whom two years after LC, multiple hard nodules were incidentally found in the omentum during cesarean section raising the possibility of a metastatic disease [25]. Kakaty et al. reported a 57-year-old female in whom, after eight years of LC, evaluation of port site hernia by imaging led to the discovery of an incidental mesenteric lump which was provisionally labeled as malignant till histopathology cleared the diagnosis [26]. In a case report presented by Kim et al., a 59-year-old male, five months after LC, suffered from right-sided constant abdominal pain [27]. Imaging revealed a retroperitoneal mass mimicking the features of sarcoma. On exploration, the mass was found to be a foreign body granuloma with an abscess and retained gallstones in the subhepatic area.

Like other questionnaire-based research, the current study has its share of limitations. It is based on self-reported practices which may differ from the actual practices of surgeons in real-life settings. Moreover, the study included only Al Qassim province which limits the generalizability of the study findings to other provinces of Saudi Arabia. However, since this is the first study on the topic in Saudi Arabia, it is anticipated that this study will stimulate more extensive research and serve as an aid in spreading awareness about spilled gallstones during LC.

## Conclusions

Raising awareness of spilled gallstones after laparoscopic cholecystectomy is necessary since the study found knowledge gaps on the range of potential problems and the timing of their clinical presentation. It is necessary to standardize the procedures for managing spilling gallstones, documenting them, and gathering patient data. Every incidence of this nature needs to be documented explicitly in the operation notes and carefully explained to the patients.
